# Progress in Psychiatry

**Published:** 1901-05-04

**Authors:** 


### Progress in Psychiatry.

(*Continued from page* 318.)

**Mental Disorders following Influenza.**—The effect of influenza on the brain and nervous system is definite and sometimes severe. The special cerebral troubles following influenza, especially in predisposed subjects, are as follows: neurasthenia, insomnia, delirium, neuralgia migraine, nervous deafness, epileptic attacks, and various cerebral palsies. Influenza may give rise to insanity in neurotic subjects. Persons who have been previously insane but have recovered may relapse after an attack of influenza. Among those prone to mental derangement from influenza are persons at the critical periods of life, such as the puerperium and the menopause, and those who from alcoholic, renal or other disease are the subjects of arterial degeneration (arterio-sclerosis and atheroma). Like alcohol, the influenzal toxins leave the nervous tissues weaker after two or more attacks, and thus predispose the brain for derangement under other forms of stress. An epidemic of influenza leaves the community of sufferers weaker for future contingencies.

A patient suffering from influenza and while in the febrile stage, or in the stage of convalescence, may contract acute delirium (acute delirious mania) of a very grave character. The typical simple psychosis which follows influenza is marked by the symptoms of neurasthenia or melancholia, and where the latter condition prevails the possibility of suicide should be always borne in mind and guarded against. Insane patients who have for years been free from acute mental disturbance may have a recurrence of acute cerebral disturbance with influenza, and in the chronic vesaniæ recrudescences of mania and melancholia are not infrequently produced by influenza. Insanities marked by persecutory or expansive delusions are frequently made worse by influenza, and may even pass into a condition of acute dementia marked by apathy, stupor, and general mental deterioration. The senile brain is liable to succumb to influenza, especially if arterial degeneration is present, and any of the milder senile psychoses are liable to pass into graver forms of incurable mental degeneration after the incidence of influenza. In neuropaths and psychopaths who are not sufficiently deranged in mind to need sequestration or asylum treatment, the occurrence of influenza may lead to crimes and acts of medico-legal gravity.

In a special monograph[7,8] devoted to influenza and its sequelæ, the late Dr. Althaus enumerates the following post-influenzal psychoses, viz., delirium, mania with fever, delirium tremens, neurasthenia, hypochondriasis, melancholia, homicidal impulses, and paralytic dementia (general paralysis of the insane). The milder nervous disturbances of influenza, which patients otherwise healthy and robust may exhibit, are as follows:—Languor and depression of spirits, apathy and want of interest in surroundings, mental irritability, insomnia and cephalalgia, backache and neuralgias which sometimes simulate the lightening pains of tabes, melancholia and slight suicidal ideas. Other bodily troubles include constipation and flatulence (except in the gastro-intestinal form of influenza, in which there is diarrhœa and gastro-enteritis), and occasional hyperpyrexia. Gibson (quoted by Althaus) mentions the case of a man with a temperature of 109° Fahr., and a woman with the same temperature in influenza. In all such hyperpyrexial cases there is grave cerebral disturbance.

In a discussion on influenza as it affects the nervous system,[79] which took place at the meeting of the British Medical Association at Ipswich (August, 1900), Dr. Judson S. Bury, of Manchester, stated that cases in which the nervous system was attacked by influenza may be broadly separated into two groups. In one group are placed the nervous diseases which develop during, or shortly after, the febrile stage, and are sometimes the sole representatives of the effects of the influenzal poison, the best examples of this being meningitis and hæmorrhagic encephalitis. There is evidence that in these diseases the brain tissue is directly attacked by the bacilli of influenza. In the second group are placed the nervous diseases which usually occur after the influenzal attack has subsided—as for example, neurasthenia and multiple neuritis. Here it is assumed that the toxins produced by the bacilli are more dilute, or less virulent than in the first group. This division is not, of course, strictly a hard and fast one, because some of the effects of the "diluted poison" may be observed during the febrile stage, while cerebral inflammatory changes may occur at a later period; still, from a clinical standpoint, the division is a convenient one.

The following case of hæmorrhagic encephalitis of influenzal origin is reported by Dr. Bury. The patient was a man, aged 31 years, a clerk, in good health. On Monday morning he went to business as usual, but complained of headache, looked ill, and could not attend to his work properly. He went home and took to bed. On the second day he was unable to eat any breakfast or dinner. On Wednesday he was noticed to be drowsy. His temperature was 102° Fahr., and his pulse 110. During the evening he grew comatose, and passed his urine and fæces in bed. On Thursday evening he was worse, the temperature 105°, the pulse 120, and he was quite unconscious and unable to swallow food. His limbs were quite rigid, the skin was dry, and occasional twitchings of the arms and legs were noticed. The knee-jerks were present. On Friday, the fifth day of illness, he seemed to rally a little, and opened his eyes when spoken to. On Saturday he could swallow a little, the rigidity was less marked, he could put out his tongue and tried to speak, but could not get out any words. On Sunday he grew worse, became comatose, and died at night. At the necropsy, made about eight hours after death, the cerebral sinuses were found engorged with blood, the dura mater was tense, the whole cortex was of a bright red colour, with a few scattered yellowish white patches. The pia-arachnoid vessels were greatly engorged. The patches consisted of fibrino-purulent exudation, especially over the anterior half of the right cerebral hemisphere. On section the long cortical arterioles were intensely hyperæmic, the white matter presented scattered hæmorrhagic points, and several hæmorrhagic patches were met with in the frontal lobe of the right hemisphere. The pia-arachnoid meshes were distended with inflammatory exudation. The lymphatic, and especially the perivascular, spaces were distended and occupied by networks of fibrin, leucocytes, and many large proliferating cells derived from the advertitial sheathes of the blood-vessels. Some vessels contained thrombi. The same changes were seen in the nutrient arteries (basal vessels) of the brain. In many instances hæmorrhages had occurred into the perivascular spaces, and even into the surrounding brain substance. The pericellular sacs were distended. Small micro-organisms (cocci) were present in the exudate in sections stained by Lœffler's and Gram's methods. The presence of Pfeiffer's bacilli in the brain tissues was doubtful. It is interesting to note that the other causes of hæmorrhagic encephalitis—itself a rare lesion—are epidemic cerebro-spinal meningitis, ulcerative endocarditis, and acute delirium. Purulent meningitis may be set up by influenza, and Fraenkel records three such cases occurring in infants and children, where cultivations from the pus showed the presence of influenza bacilli. Foa has recorded a case of disseminated hæmorrhagic myelitis occurring in influenza. As regards the nerves both neuritis and neuralgia may be present, and in this connection the occurrence of retro-bulbar neuritis (of the optic nerve) should be specially mentioned. The paralyses which are due to lesions of the ponto-bulbar nuclei or of their nerves are of great interest, because they illustrate one of the striking peculiarities of influenzal toxins, viz.: the erratic distribution of the palsies. Thus, while, as in diphtheria, there may be paralysis of accommodation associated with paralysis of the palate, there is a greater variety as regards the grouping of the muscles affected with paralysis. Thus we meet with isolated paralysis of the superior rectus, or of the internal or external recti, transitary dilatation of one pupil, and intermittent paralysis of accommodation. Similarly the facial or hypoglossal nerve may be affected. Recovery from such paralysis is in such cases the general rule, whence it has been assumed that the morbid changes affecting the cranial nerves or their nuclei are of a slight and transitory character.

Dr. Thomas Buzzard,[81] of the National Hospital for the Paralysed and Epileptic, was of opinion that existing terminology was inadequate to cover and include the various multiform nervous lesions of influenzal origin. In many instances the influence of the toxin was extensive, embracing widely separate groups of neurons, and it was quite impossible to include the cases in any recognised category of diseases of the nervous system. In many cases he had been struck with a similar bizarre distribution of symptoms in disease of the nervous system of syphilitic origin, while Medin, of Stockholm, and others had also recently called attention to the fact that in epidemics of so-called infantile paralysis, whilst the brunt of the attack might fall perhaps on the anterior cornua of the spinal cord, there would in many cases be concomitant localised paralysis of a cranial nerve, symptoms of encephalitis, or lesions of the lateral column—indications, in a word, of scattered focal lesions in various parts of the nervous system. He thought this peculiar character, in the circumstances, gave a very strong support to the view that the grave lesions observed in influenza, like those in syphilis and in epidemics of infantile paralysis, were dependent upon the effect of toxins on the nervous structures.

According to Kraepelin, Ladame, Hurd, and other Continental and American observers, the most frequent form of psychosis after influenza is acute mental confusion with hallucinations (hallucinatory delirium of Mendel), this condition subsiding at a later stage into agitated melancholia or grave neurasthenia. Hypochondriacal delusions may occur if mental enfeeblement persists. Stuporous states develop with less frequency. Generally recovery takes place unless there is a strong existing predisposition to insanity. The treatment consists of rest, enforced feeding, tonics containing strychnine and quinine, and small doses of alcohol.

[78] Althaus, Influenza: its Pathology, &c., 2nd edit., 1892. [79] Brit. Med. Journ., 29th September, 1900. [80] Brit. Med. Journ., loc. cit. [81] Ibid.

